# On the Nature and Treatment of Stomach and Renal Diseases; Being an Inquiry into the Connexion of Diabetes, Calculus, and Other Affections of the Kidney and Bladder, with Indigestion

**Published:** 1843-10

**Authors:** 


### ART. XVI.

*On the Nature and Treatment of Stomach and Renal Diseases; being an Inquiry into the Connexion of Diabetes, Calculus, and other Affections of the Kidney and Bladder, with Indigestion.* By WILLIAM PROUT, M.D. F.R.S. &c. Fourth Edition, revised.—*London*, 1843. 8vo, pp. 593.

THE author's own preface to this new edition of his valuable work, will best explain the views with which he has prepared it:

"The present edition is essentially the same as the last. The chief alteration consists in the arrangement of the introduction, which now constitutes a Third BOOK. As an introduction, this part of the volume was already too long; and as I could not add a few necessary remarks without rendering it still more unwieldy, I was induced to make the change in question.

"Since the third edition was published, Professor Liebig's treatises on Vegetable and Animal Chemistry have made their appearance, and attracted no little notice. Some of the views advanced by this distinguished chemist in his last work are the same I have long advocated. Others of his views are directly opposed to mine, and seem to me to be neither susceptible of proof, nor even probable. The *practical* nature of this volume, however, precludes all controversy, particularly on matters of no practical utility; and I allude to the subject, chiefly for the opportunity of observing, that having in the following pages stated my own opinions without reference to Professor Liebig, I leave it to the public to decide whether he or I have most nearly approached the truth.

"There is another point also connected with this part of the subject, which requires a few remarks. I have purposely omitted the formulæ, now so much in fashion among chemists, not only because I consider them clumsy and unphilosophical as conventional expedients, but because I am satisfied that very few, if any, of them, represent the true constitution of organized substances. A grand clue to many chemical phenomena will be found among the multiple relations of what are termed the atomic weights of bodies. After nearly thirty years, chemists have reluctantly admitted the existence of such relations among the four constituent elements of organized bodies. Another generation, I have no doubt, will recognize and admit the important consequences to which these relations lead." (pp. vii-viii.)

On the three paragraphs composing this preface, we shall offer a few remarks before proceeding further. We quite agree with Dr. Prout in the desirableness of converting his introduction into a book, on account both of its bulk and its importance; but we should have thought it better to allow it to retain its previous position at the commencement of the volume, instead of transferring it to the end. The student must necessarily acquaint himself with the physiological or normal actions of the body, or of any portion of it, before he can rightly comprehend its pathological or abnormal conditions; and in Dr Prout's treatise this order appears to us to be particularly required, since so large a part of his views on the disordered action of the assimilating and secreting organs, are positively incomprehensible without a previous acquaintance with his ideas of their physiological connexion. We strongly recommend the readers of the present edition, therefore, to reverse the author's arrangement, and to study the last book first.

We also quite agree with Dr. Prout in the undesirableness of introducing controversy into a practical work of this kind; nevertheless, we think that it would not have been amiss to have inserted a few references to Liebig's peculiar opinions, as a guide to the student in the comparison of them with Dr. Prout's views. A good deal of trouble might have been thereby spared to those who, like ourselves, desire to become fully acquainted with the points at issue between these two distinguished chemists. We trust that Dr. Prout may see the desirableness of publishing, in a separate form, and with more amplification, his opinions on these controverted topics, particularly specifying the evidence on which his own views are founded. Professor Liebig's work is almost entirely of an argumentative kind; the data on which his reasonings are founded are for the most part specified; and thus every reader, possessing a competent knowledge of the subject, can form his own opinion—from his knowledge of the probable truth or error of the data, and from his estimate of the logical precision of the reasoning,—as to the value of the conclusions drawn and set forth by the author. In Dr. Prout's treatise, on the other hand, there is more of assertion, and less of even attempts at proof, the data being, for the most part, locked up in the author's own laboratory; and until Dr. Prout shall see fit to give them to the public, he must be content to have his opinions freely questioned, and the accuracy of his conclusions suspected. We earnestly hope that he may be induced to publish the results of his laborious inquiries, in such a form as may obtain for him that rank amongst organic chemists, to which we feel assured that he is justly entitled.

We are glad to find that Dr. Prout so fully agrees with the opinion we expressed in our last Volume (p. 507), in regard to the fashionable system of *formulæ*. Our remarks were addressed to what we conceived to be their misuse. To their great utility, in a great number of cases, we freely bear testimony. But we cannot place the full confidence in them which some entertain, for the following reasons—1. The atomic weights or combining equivalents of many of the simple or elementary substances are far from being indubitably ascertained. Take that of carbon for example, a correct determination of which is so important in regard to others. Almost every chemist has been in the habit of estimating this at a fraction above *six*. The result obtained by Dr. Turner in 1833, from a series of experiments of which the elaborate accuracy commanded for them the highest regard, seemed to fix it positively at 6·12. Yet Dr. Prout tells us (p. 556, note) that he long ago settled to his own satisfaction, by numerous most careful experiments, that the combining weight of carbon is neither more nor less than 6; this conclusion is the one recently arrived at by Dumas, after a very elaborate series of experiments, and it seems to be gaining ground amongst chemists. We do not offer an opinion as to the correctness of either of these numbers; but we simply say that, until the claim of one of them to adoption is placed beyond all question, no great confidence can be placed in formulæ.—2. Notwithstanding the great improvements which have been made during the last few years, especially by Liebig, in the analysis of the organic proximate principles, there is still a great degree of uncertainty in regard to the exactness of the results obtained. "I know at present," says Dr. Prout, (loc. cit.) "of no apparatus, or means of operating, capable, when azote is concerned, of unequivocally deciding about the presence or absence of *one proportion* of hydrogen or even of oxygen in a complicated body. Liebig's analytic apparatus was in effect tried by me twenty years ago; and for rude approximations it answers very well; but it is not, in my opinion, at all adapted for obtaining very accurate results." Many of those who can reason most acutely upon formulæ, and show to a nicety how every atom of oxygen, hydrogen, carbon, and nitrogen, is disposed of, in the disintegration of albumen and gelatin, are unaware how much room there still is for questioning the results of the analyses on which those formulæ are based.—3. Even supposing that the absolute number of atoms of each of the elements making up an organic compound, were ascertained by a perfect analysis, still the formula must be considered as far from representing the real state of combination. We have no reason to believe that, in any instances, four sets of atoms unite with each other in the manner which we should be thus led to suppose; and we cannot but think that the real constitution of protein is not represented by the formula O. 14+H. 36+C. 48+N. 6, one whit better than that of sulphate of potass would be by the formula S.+K.+O. 4. The inorganic chemist well knows that the constitution of the latter, according to the usual mode of representing it, is (S.+O. 3)+(K.+O.); or according to the present views of the constitution of salts, as consisting of a base directly united to a compound radical (S.+O. 4)+(K.) Each of these formulæ represents a *fact* in regard to the arrangements of the elements of which the compound is made up; the first expresses that it is formed by the union of sulphuric acid and potash, into which it may be again decomposed; the second expresses the doctrine, now generally received amongst chemists, that, when the combination of these two bodies is effected, their elements are newly arranged, so that the base potassium is united with the compound radical sulphatoxygen. Now until we are able to resolve the complex formulæ, by which the constitution of the quaternary organic compounds is now expressed, into other and simpler ones, which shall express, with some appearance at least of probability, their real constitution, we think that Dr. Prout is fully justified in his objection to their use, as expressions of facts, more particularly in consequence of the abuse to which the employment of them is evidently liable. That they may be of great utility as *guides to research*, we freely admit; but, like many similar expedients, their use is temporary only; and we cannot regard deductions from them as satisfactory, until the conversions which they are supposed to indicate have been either effected in the laboratory of the chemist, or have been shown, by pretty clear evidence (physiological and pathological), to take place in the living body.

In our critical review of the former edition of Dr. Prout's treatise, we entered into a pretty full examination of his peculiar views; and the following was our general conclusion :

"We acknowledge and have pride in bearing testimony to the high qualifications of our countryman in the branch of pathological inquiry based upon chemical facts; we recognize the comprehensive sagacity of his speculations, and have respect for the patient zeal with which he has toiled to erect upon these a stable system. But we fear the time for such systematizing has not yet come; and although all speculations on the subject are seductive in themselves, and doubly so when emanating from an individual of Dr. Prout's eminent skill in the department of chemical physiology, it cannot, we think, be denied that in the existing unformed and vacillating state of organic chemistry, they sin essentially in being established on a most unsound basis. Nor can we avoid entertaining some solicitude as to the results of their propagation, which to us appears likely to betray minds of inferior order into mere extravagances. For these, however, Dr. Prout is not fairly answerable; and should his doctrines—when the frail embryo science on which they are based has reached healthy maturity—be recognized as true, he must almost take rank with those highest intelligences, whose energy has outrun the scientific apprehension of their times. But meanwhile Dr. Prout has neither done his doctrines, himself nor his readers justice in not explicitly stating the foundation for and manner of verifying (so far as he is acquainted with these himself,) his presumed results." (Vol. XI. p. 363.)

We have learned with regret that our criticism, candid and honest as we maintain it to have been, gave pain to the eminent author of the work; and we have reverted to it on this occasion, with the earnest desire to repair, so far as lies in our power, any injury we may have unintentionally inflicted by errors of omission or of commission. When a man of high repute puts forth his opinions *ex cathedrâ* on important practical questions like the present, it behoves those who occupy a position like ours, to exercise (if that be possible) a double measure of their usual critical acumen; in order that they may prevent errors, sanctioned by the authority of a great name, from gaining that prevalence which, when brought forward by those of less note, would have comparatively little injurious influence. It was on this account that, without any desire to depreciate what we imagined to be the universally acknowledged merit of Dr. Prout's labours, we addressed ourselves to the consideration of those points, on which, as we then conceived, and still believe, he had failed in establishing the positions he advanced. But, if disposed to criticise our own criticism, we should now say that, without admitting it to contain any serious error of commission, it ought to have contained a higher tribute than it contains, to what we may regard as the general and distinguishing merit of Dr. Prout's treatise,—the object to which his whole life has been devoted,—namely, the exposition it contains of the important connexion between a large number of disordered states of the urinary secretion (and these the most important) and disordered states of the processes of digestion and assimilation. For the clear and positive idea of this connexion, not only in one or two diseases, but in regard to a large number,—an idea, too, of the most extensive application in regard to the whole physiology and pathology of assimilation and excretion,—we believe that we are mainly indebted to Dr. Prout. And whether the results of future inquiries shall or shall not confirm his *particular* doctrines, we desire to record here our deliberate conviction that the *direction* was first given to those inquiries by Dr. Prout, and that the physiologist, the pathologist, and the practitioner, ought therefore to feel themselves lying under a debt of gratitude to him, which no errors or imperfections in the details of his labours can efface.

With the view of more strongly impressing Dr. Prout's merits, in this respect, on the minds of our readers, we shall offer them a brief summary of his general doctrines, and of their most important applications; pointing out some of the leading differences between his views and those of Liebig.

The conversion of alimentary materials into organized tissue is regarded by Dr. Prout as divisible into two stages, to which he gives the name of *primary* and *secondary* assimilation. Under the term *primary* assimilation he includes all the changes which the food undergoes, from its entrance into the stomach up to its conversion into the materials of blood,—or in other words, up to the time of its possessing an organizable condition. By *secondary* assimilation he designates the conversion of the organizable materials of the blood into organized tissue; and under the same head,—not we think without some incongruity,—he classes the subsequent *destruction* of these tissues, and their resolution into other compounds, which are destined to be excreted.

Dr. Prout does not regard the digestive process as one of simple *reduction* and *solution;* but believes that an actual *conversion* of one form of alimentary matter into another may be effected by it.

"Two, indeed, of the chief materials from which chyle is formed, namely the albuminous and oleaginous principles, may be considered to be already fitted for the purposes of the animal economy, without undergoing any essential changes in their composition; but the saccharine class of aliments, which form a very large proportion of the food of all animals, except those entirely subsisting on flesh, are by no means adapted for such speedy assimilation. Indeed, one or more essential changes must take place in saccharine aliments, previously to their conversion either into the albuminous or the oleaginous principles.........We cannot trace the conversion of sugar into albumen, because we are ignorant of the relative composition, and of the laws which regulate the composition of these two substances. The origin of the azote in the albumen, is likewise at present unknown to us, though in all ordinary cases it seems to be appropriated from some external source. That the oleaginous principle may be converted into most, if not all, the matters necessary for the existence of animal bodies, seems to be proved by the well-known fact, that the life of an animal may be prolonged by the appropriation of the oleaginous and other matters contained within its body.........Under ordinary circumstances, then, the converting powers of the stomach must essentially consist of the three kinds mentioned, viz. the conversion of saccharine aliments into albuminous and oleaginous principles; and the conversion of oleaginous into albuminous principles." (pp. 470-1.)

Now we consider it to be a question of the very highest moment, to ascertain how far this doctrine of *conversion* is well founded. Our readers are probably well aware that it is admitted by Liebig and his followers only, in regard to the *azotized* and *non-azotized* articles of food considered separately;—that is, they consider albumen as capable of transformation into gelatin, horny matter, or any other azotized compound, and also, by a completely new arrangement of its elements into oleaginous matter; and again, they regard the saccharine principle (including *starch* in all its forms) as capable of transformation into the oleaginous; but they completely deny the possibility of the transformation, under any circumstances whatever, of the saccharine or oleaginous principles into the albuminous. By Dumas and his followers, (who have been recently carrying on a hot controversy with Liebig on this question,) it is denied that even the conversion of saccharine into oleaginous principles ever takes place in the animal body. The saccharine principles are by them regarded as entirely disposed of by respiration, or by conversion into lactic acid; and the oleaginous principles as being carried out of the system by the biliary and other excretions, as well as by respiration, as fast as they are introduced into it,—unless deposited as fat.

Although these doctrines of the continental chemists have been received as valid by many high authorities, we think that much more extended inquiry is necessary, before they are entitled to rank as ascertained facts; and we hold, with Dr. Prout, that although the conversion of saccharine or oleaginous matter into albuminous may not be a part of the ordinary process of nutrition, when an animal ingests a proper proportion of the several alimentary principles, it *may* take place *to a limited extent*, when such a conversion is required for the supply of the wants of the system. It seems to us to be the duty of those who deny *in toto* the possibility of this, to explain the following facts, or to show that they are incorrectly stated. 1. However large may be the proportion of saccharine matter in the food, it is not to be detected in the healthy state, either in the chyle or the blood. In what state, then, is it received into the circulation? By Liebig and his followers it will be said that it is converted into oleaginous matter. Granted; but then, 2. By microscopic-chemical examination of the chyle it seems clearly ascertained, that the proportion of oleaginous matter in the chyle, which is usually very large in the peripheral lacteals, gradually diminishes; and that the proportion of fibrin, and probably that of albumen also, gradually increases, until the composition of the fluid approaches more nearly to that of the blood. This seems to us to indicate the continuance of the converting process, which, according to Dr. Prout, commences in the stomach and duodenum. The only positive evidence which he adduces to this effect, however, is contained in a note (p. 504); in which he states that he has "constantly found albumen developed in abundance in the duodenum of animals, whether the food contained azote or not." Now we would earnestly request Dr. Prout to give to the world the precise data on which he founds this statement; and to repeat his experiments, if requisite, in such a form as to leave no doubt of the absence of azote in the food, and the presence of albumen in the duodenum. At present they lie open to the objection, that the articles likely to have been employed, such as starch, sugar, &c., may have contained a small quantity of azote. Yet we think that by far too much importance has been attached to this circumstance by the chemists previously referred to. For the question is *not*, whether rice, potatoes, cassava, and other forms of starchy matter that constitute the staple food of many races of men, contain azote, but whether the azotized principle exists in them in an amount sufficient to supply the wants of the system. For ourselves, we cannot believe that this is the case.

There is another form of the nutritive process, which we consider of great consequence in determining this question, and on which it would not be difficult to make conclusive experiments; we refer to that which presents itself in hive-bees. The case has been already adverted to by Liebig, as proving that the saccharine principle is capable of being converted into the oleaginous; since bees are well-known to be able to produce wax, when shut up in their hives, and fed with pure sugar. But we wonder that it did not occur to Liebig, that, by parity of reasoning, the saccharine principle must be capable of conversion into the albuminous. It is quite true that, in the expressed juice of the sugar-cane, maple, &c., there is present a small quantity of azotized matter, sufficient in amount to become a very active *ferment*; but it is the first object of the manufacturers to get rid of this; and we believe that in the finer kinds of brown sugar, and in all refined sugar, it would be very difficult to find a trace of azote. If bees feed upon this material alone, they will not only make honey and wax, but elaborate from it the materials of their muscular fibre and other tissues, the question must be regarded as decided that animals can convert saccharine into albuminous matter; although it still remains an open question, to what extent this power may exist in the higher classes. We believe it to be a fundamental error in the physiological chemistry of Liebig, that he has studied animal life under a very few only of its phases; and that he has consequently been led to conclusions which a more extended survey shows to be invalid. We pointed out this formerly, in regard to his doctrine of the connexion between the biliary secretion and the respiratory process, which we showed to be completely inapplicable to insects and mollusca, (vol. XIV, p. 514;) and we have his own authority for stating that this view of the case had never occurred to him. In like manner, we cannot think that he would have advanced this doctrine of the absolute inconvertibility of the saccharine into the albuminous principle, if he had pondered upon the vast number of the insect tribes, and these, too, including the insects most distinguished by muscular activity, and therefore requiring (on Liebig's own theory) the largest amount of azotized nutriment, which derive their whole support from the saccharine juices which they imbibe from flowers. It is quite possible that these juices may contain azote; but it must be shown that they contain enough to replace the *waste* that must take place in the tissues of these active little beings.

Regarding the source of the azote thus added, Dr. Prout's opinion seems to us well worthy of consideration:

"The azote may, in some instances, be derived from the air, or *generated* (?) But my belief is, that, under ordinary circumstances, much of the azote employed in the assimilation of saccharine matter is furnished by a highly-azotized substance secreted from the blood, chiefly into the duodenum; and that the portion of the blood thus deprived of azote is separated from the general mass of the blood, either by the stomach in the form of lactic acid, or by the liver, as one of the non-azotized constituents of the bile; and that the lactic acid and non-azotized substance thus separated are ordinarily excrementitious." (p. 470, note.)

By Liebig it is supposed that one of the great purposes of the saliva is to carry down a quantity of atmospheric air, for the supply of *oxygen* supposed by him to be required in the process of digestion. Dr. Prout points out that *azote* may be thus introduced:

"The atmospheric air involved during the mastication and insalivation of their food is very probably another source of azote to vegetable feeders. Indeed this involution of azote may be considered as *one* of the great objects of mastication, &c., which is almost peculiar to animals chiefly subsisting on saccharine matters." (p. 504, note.)

We have dwelt the longer upon this topic, both on account of its intrinsic importance, and because we desire to uphold what we believe to be the correct views entertained by Dr. Prout, against the untenable assumptions (so at least we at present regard them) of continental chemists.

In abnormal states of the *converting* process, Dr. Prout looks for the cause of some of the most troublesome forms of indigestion, and for the foundation of the excess of sugar in the system, in diabetes. This excess of sugar (which has been proved beyond all doubt to exist in the blood) may be derived in part from the want of power to *convert* saccharine aliments, which are therefore absorbed in their original state. It would also seem that conversion of albuminous or gelatinous principles into the saccharine may take place in the stomach, for it has been ascertained that sugar is formed in the stomach, even when the food is exclusively animal. Hence it probably is, that not even an exclusively animal diet is successful in preventing the production of sugar; and that, by the ingestion of a small quantity of saccharine matter, which may act as a kind of *ferment*, the quantity thus produced is liable to be very greatly increased. The practical remark of Dr. Prout, "I have known the use of a few saccharine pears undo, in a few hours, all that I had been labouring for months to accomplish," is probably familiar to most of our readers. We regret not to find, under the head of the dietetic treatment of diabetes, any reference to the gluten-bread, which has been introduced, since the publication of the previous edition of Dr. Prout's treatise, by M. Bouchardat—with the view of giving that variety to the diet, which patients long restricted to animal food almost imperatively demand, without doing injury by admitting farinaceous (saccharine) matter into the system. The efficacy of the complete restriction of the diet to azotized matter, which can be thus maintained for any length of time, (a thing extremely difficult of accomplishment with *meat* alone, owing to the absolute *craving* of the patient for something else,) has been spoken of in high terms in Paris; and it has been tried with success in London. As few if any persons have larger opportunities of making trials of this kind than Dr. Prout possesses, we beg to recommend the subject to his attention, and hope to be favoured with the results of his experience.

The power of converting the saccharine principle in the stomach may be *deranged* as well as suspended; and this derangement may give rise to the production of the lactic or oxalic acids in abnormal amount, occasioning their absorption into the blood and the numerous train of symptoms consequent thereon, which we need not now enumerate. We believe that Dr. Prout is perfectly correct in tracing the first of these, at any rate,—and probably the second, in a large number of instances,—to derangement of the *primary* assimilating processes, and especially to that of saccharine conversion.

In regard to the *secondary assimilating* process (strictly so called), our knowledge is necessarily more limited. It is quite possible that, in consequence of an imperfect elaboration of the organizable materials, or an insufficient demand for them, the chief constituents of the blood may be changed into excrementitious substances without even passing through the form of organized tissue; and we have elsewhere endeavoured to show that this *must* take place, in regard to azotized matter, whenever the quantity of it which is absorbed considerably exceeds the quantity that can be organized. But as to the products which are liable to be formed under such circumstances, we have no certain knowledge. It appears to us that Dr. Prout's idea, that the albumen of the blood may, by a derangement of the secondary formative assimilating process, be converted into urea and a saccharine principle,—instead of being normally converted into gelatin,—is rather an assumption than a proved fact. It would seem probable, however, that the results of this decomposition should be similar to those which substances of the same constitution produce by their disintegration, *after* they have passed through the form of organized tissue, in the process termed by Dr. Prout (rather incongruously as it seems to us) *destructive assimilation ;* and by Liebig, *waste,* or *metamorphosis.* In his opinion, the metamorphosis of the *albuminous* principles gives origin to lithic acid ; and that of the *gelatinous* to urea and a saccharine principle, usually lactic acid. The data upon which this opinion is founded are not given, for the reasons already referred to ; but we would again urge upon Dr. Prout the propriety of making them public, if it be only to show that physiological and pathological chemistry has not been neglected in this country to the extent usually supposed, and that Liebig's doctrines are not to be received without the consideration of a much larger number of circumstances than their author seems to have taken into account. For ourselves, we must frankly say that we do not think the distinction which Dr. Prout attempts to establish can be sustained; for this among other reasons,—that, as there is great reason to believe, that the metamorphosis of the albuminous tissues usually takes place (in consequence of muscular exercise) much faster than that of the gelatinous,—we can scarcely imagine the large proportion of urea found in the ordinary urine of the mammalia to be generated from the gelatinous tissues only; nor can we suppose all the uric acid generated in the urine of birds and reptiles to be the produce of their albuminous tissues only. We hope that the researches of chemists, directed to this object, may be successful in throwing light ere long upon this very important question.

Derangements of the destructive assimilation or metamorphosis of tissues are among the most fertile sources of disordered conditions of the excretions; and although Liebig has recently directed attention to these in such a prominent manner, and with such a show of reason, as to have commanded the implicit assent of a large number of followers, yet it would be doing great injustice to Dr. Prout if we did not assert our deliberate conviction that, however much the results obtained by the illustrious professor of Geissen differ from his (and here the question remains open), the path of inquiry which he has pursued is essentially the same with that first opened by our illustrious countryman, and for a long time pursued by him alone.

We shall conclude this article by a notice of some peculiar views of Dr. Prout's, which are frequently adverted to but nowhere distinctly stated, in the volume before us; but which are more clearly laid down in his Bridgewater Treatise. Dr. Prout considers that, after the *death* of the tissues, their *metamorphosis* into excrementitious compounds, resembling those of inorganic matter, does not necessarily take place immediately, but that a portion of them may be again taken into the system and made use of as materials for its nutrition ; in other words, that an animal may partly live upon its own tissues. This he supposes to take place by a kind of secondary digestion, occasioned by the liberation of an acid fluid in the capillaries; and he considers that the process of *conversion* may take place *there* as well as in the stomach, so that oleaginous matter (as fat) may be changed into an azotized compound fit for the nutrition of the albuminous tissues. The *mode* in which this kind of change is effected must be admitted to be in a great degree hypothetical; but that such a change does occur we think there is a strong probability. The objection has been advanced that it is absurd and contrary to all sound physiology to suppose that organic matter which has once lost its vitality can be again taken into the system and enter into the composition of organized tissue; but it seems to have been entirely forgotten by the objector that all the aliment of which we make use is in a dead state or is rendered so during the process of digestion. There is nothing more absurd, therefore, in the idea that a man receives back into his circulation (and therefore partly lives upon) a portion of his own tissues, than that he takes in, recombines, and organizes, similar dead matter from the tissues of another animal. And the hypothesis accords remarkably well with one proposed by Dr. Carpenter, (Human Physiology, § 464-7,) repecting the use of the *lymphatic* system; namely, that it serves to take up, assimilate, and convert into the elements of blood the materials thus set free in the tissues; just as the lacteals take up, assimilate, and convert into the elements of blood the materials prepared by the digestive process. The obvious analogy between the two sets of vessels, their termination in a common trunk, their simultaneous appearance as we pass from the invertebrated to the vertebrated classes, and the very similar characters (both chemical and microscopical) of their usual contents, appear to us strong arguments in favour of this view; and if it be admitted, Dr. Prout's doctrine almost necessarily goes with it.

In taking our leave of Dr. Prout's treatise we have only to repeat our conviction that, in spite of all the faults which we formerly pointed out in it, there is so much of the highest value in its contents that no student or practitioner can be regarded as even tolerably acquainted with the subject who has not read and mastered them.

---

